# Supplementary material for: Human genomic DNA is widely interspersed with i-motif structures
Source: EMBO J. 2024 Aug 29;43(20):16. doi: 10.1038/s44318-024-00210-5 (PMC11480443; doi:10.1038/s44318-024-00210-5)
Supplement: Supplementary file 4 — Expanded View Figures [file 44318_2024_210_MOESM4_ESM.pdf]

Expanded View Figures

Figure EV1. iM immunoprecipitation (controls, repeats).

(**A**) Regions observed across replicate experiments, intersected iM regions observed after immunoprecipitation of protein-depleted purified DNA from the MCF7 cell-line protein-depleted DNA. Immunoprecipitation experimental repeats using an incubation temperature of 16 °C or 4 °C. (**B**) Regions observed across experiment repeats in DNA from two different cell-lines. Replicates for HEK293T and U2OS protein-depleted DNA from pulldowns conducted at 4 °C. (**C**) Pairwise intersection-fraction of overlap pie charts of all-vs-all experiments conducted. (**D**) Genomic view highlighting an iM structure upstream of the oncogenes *ATM*, *SIRPA*, and *TSHR*. iM regions from each cell line replicates are shown (green tracks: MCF7, brown tracks: MCF7 DNA incubated at 16 °C, purple tracks: HEK293T, blue tracks: U2OS, lower tracks: immunoprecipitation negative control input profiles). (**E**) hTelo positive control CD curve under variable pH conditions (left panel) and validation of an identified iM candidate upstream of *SIRPA* and *TSHR* by DNA synthesis following CD spectroscopy under variable pH (pH 5–8) and a temperature of 25 °C. CD Circular Dichroism.

© The Author(s)

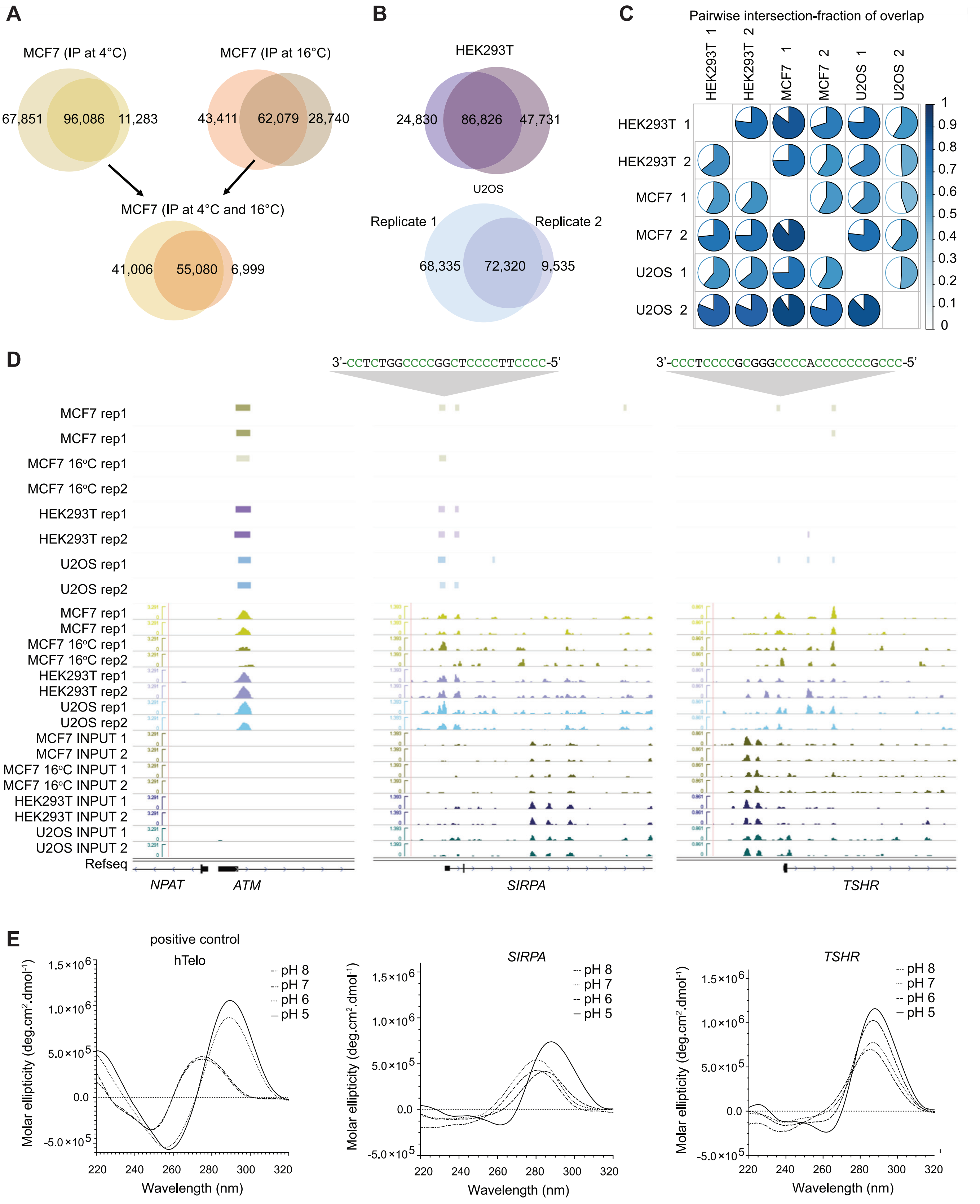
A
MCF7 (IP at 4°C)
67,851
96,086
11,283
MCF7 (IP at 16°C)
43,411
62,079
28,740
MCF7 (IP at 4°C and 16°C)
41,006
55,080
6,999
B
HEK293T
24,830
86,826
47,731
U2OS
Replicate 1
Replicate 2
68,335
72,320
9,535
C
Pairwise intersection-fraction of overlap
HEK293T 1
HEK293T 2
MCF7 1
MCF7 2
U2OS 1
U2OS 2
D
3'-CCTCTGGCCCCGGCTCCCCTTCCCC-5'
3'-CCCTCCCCGCGGGCCCCACCCCCCCGCCC-5'
MCF7 rep1
MCF7 rep1
MCF7 16°C rep1
MCF7 16°C rep2
HEK293T rep1
HEK293T rep2
U2OS rep1
U2OS rep2
MCF7 INPUT 1
MCF7 INPUT 2
MCF7 16°C INPUT 1
MCF7 16°C INPUT 2
HEK293T INPUT 1
HEK293T INPUT 2
U2OS INPUT 1
U2OS INPUT 2
Refseq
NPAT
ATM
SIRPA
TSHR
E
positive control
hTelo
SIRPA
TSHR
pH 8
pH 7
pH 6
pH 5
Molar ellipticity (deg.cm².dmol⁻¹)
Wavelength (nm)

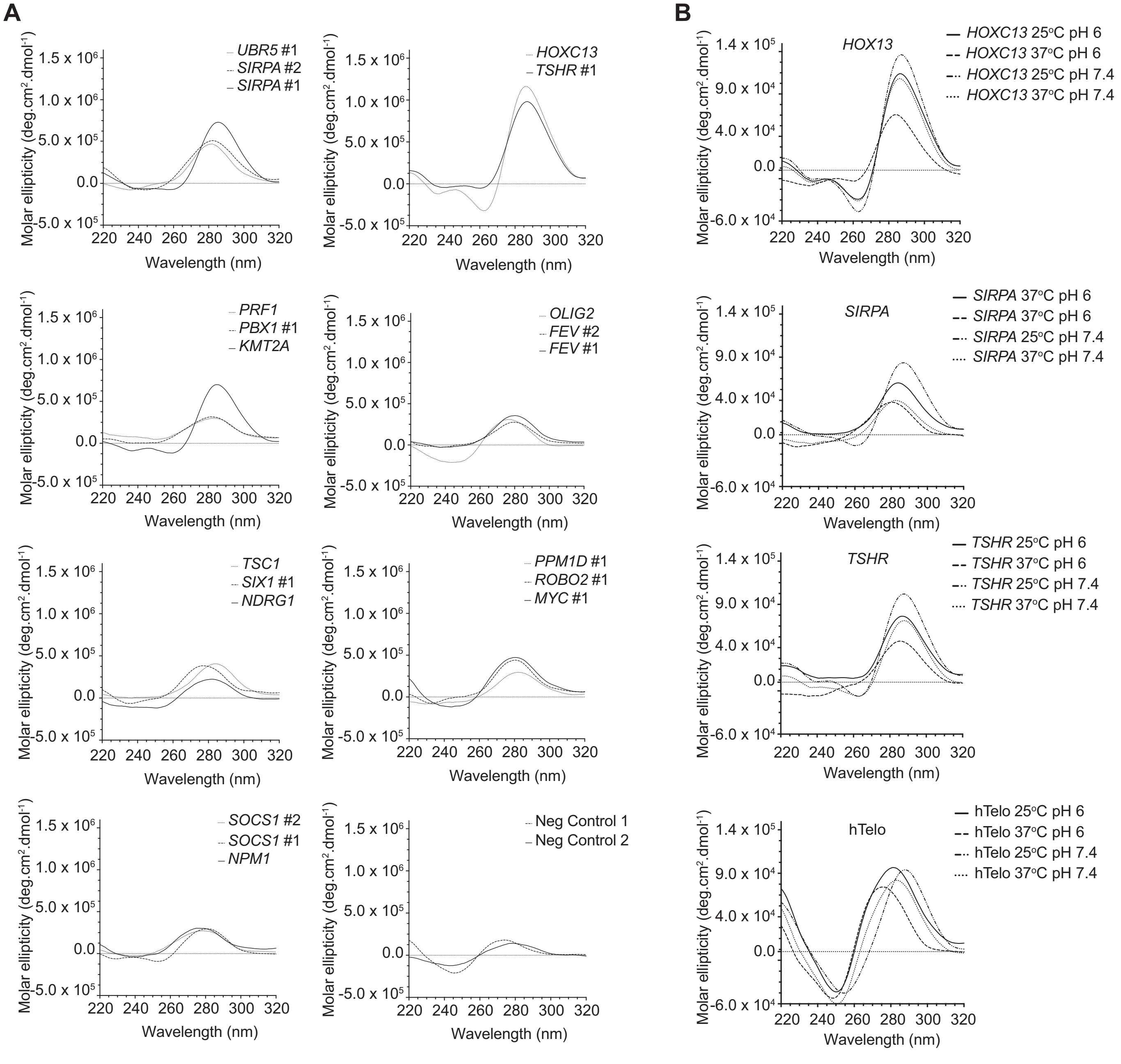

Figure EV2. Biophysical validation of iM folding.

(**A**) Validation of identified iMs by DNA synthesis and circular dichroism spectroscopy at pH 6.0 and a temperature of 25 °C of selected sequences proximal of promoter regions in known oncogenes. NC1(5′-CAGACTGTCGATGAAGCCCTG-3′) and NC2 (5′-CTAGTTATTGCTCAGCGGTG-3′) negative control sequences. (**B**) Effects of Temperature (25 °C or 37 °C) and pH (pH 6 or pH 7.4) on CD spectroscopy using identified iM regions associated to the genes *HOXC13*, *SIRPA*, *TSHR*, and the positive control hTelo sequence.

 © The Author(s)

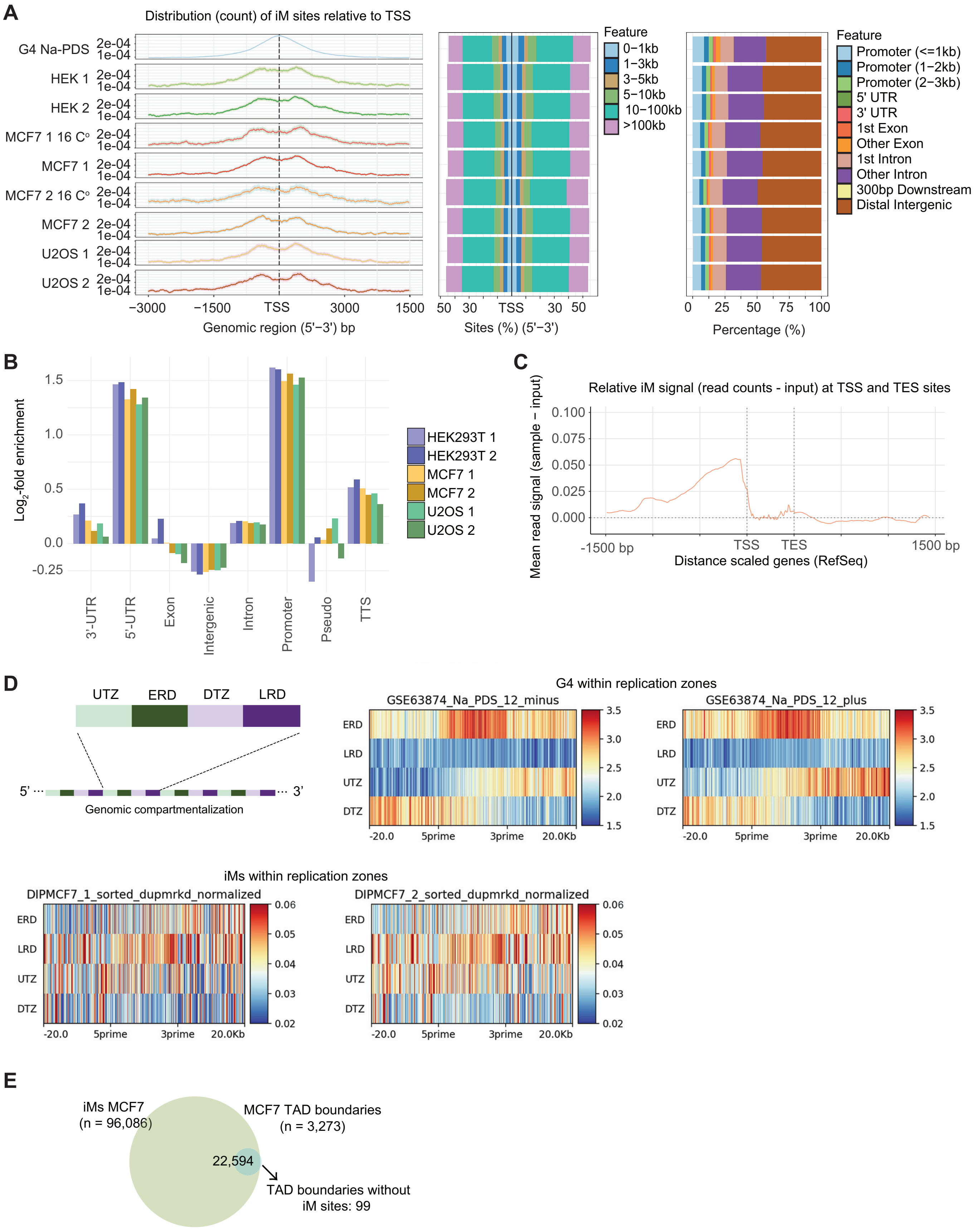
A
Distribution (count) of iM sites relative to TSS
G4 Na-PDS
HEK 1
HEK 2
MCF7 1 16 C°
MCF7 1
MCF7 2 16 C°
MCF7 2
U2OS 1
U2OS 2
2e-04
1e-04
−3000
−1500
TSS
3000
1500
Genomic region (5'−3') bp
Feature
0−1kb
1−3kb
3−5kb
5−10kb
10−100kb
>100kb
Sites (%) (5'−3')
Feature
Promoter (<=1kb)
Promoter (1−2kb)
Promoter (2−3kb)
5' UTR
3' UTR
1st Exon
Other Exon
1st Intron
Other Intron
300bp Downstream
Distal Intergenic
Percentage (%)
B
Log2-fold enrichment
1.5
1.0
0.5
0.0
-0.25
3'-UTR
5'-UTR
Exon
Intergenic
Intron
Promoter
Pseudo
TTS
HEK293T 1
HEK293T 2
MCF7 1
MCF7 2
U2OS 1
U2OS 2
C
Relative iM signal (read counts - input) at TSS and TES sites
Mean read signal (sample − input)
0.100
0.075
0.050
0.025
0.000
-1500 bp
TSS
TES
1500 bp
Distance scaled genes (RefSeq)
D
UTZ
ERD
DTZ
LRD
5' ··· ··· 3'
Genomic compartmentalization
G4 within replication zones
GSE63874_Na_PDS_12_minus
GSE63874_Na_PDS_12_plus
ERD
LRD
UTZ
DTZ
-20.0
5prime
3prime
20.0Kb
iMs within replication zones
DIPMCF7_1_sorted_dupmrkd_normalized
DIPMCF7_2_sorted_dupmrkd_normalized
E
iMs MCF7
(n = 96,086)
MCF7 TAD boundaries
(n = 3,273)
22,594
TAD boundaries without
iM sites: 99

Figure EV3. Distribution and sequences of iM regions in common genomic features, DNA replication zones and TAD boundaries.

(**A**) Distribution of genomic DNA iM structures across the human genome features. Left panels show the distribution of iM pulldown relative to all TSS regions (Refseq). Centre panels show the percentage of occupancy of iM sites relative to TSS distance. Right panels represent the percentage in relationship with the common gene body features. (**B**) $\log_2$ fold enrichment of iM regions distributed across most common genomic annotations. (**C**) Relative immunoprecipitated DNA mean signal normalised (sample-input) in relationship to RefSeq gene coordinates. (**D**) Genomic partition of replication zones (analyses based on repetition of up transition zones (UTZ), early replication zones (ERD), down transition zones (DTZ) and late replication domains (LRD); Tag count heatmaps of G4 (Chambers et al, 2015) (upper panels) and iMs (lower panels; two biological replicates shown). (**E**) Overlap of iM regions found in DNA purified from MCF7 replicates and previously reported MCF7 TAD boundaries (GSE66733).

 © The Author(s)

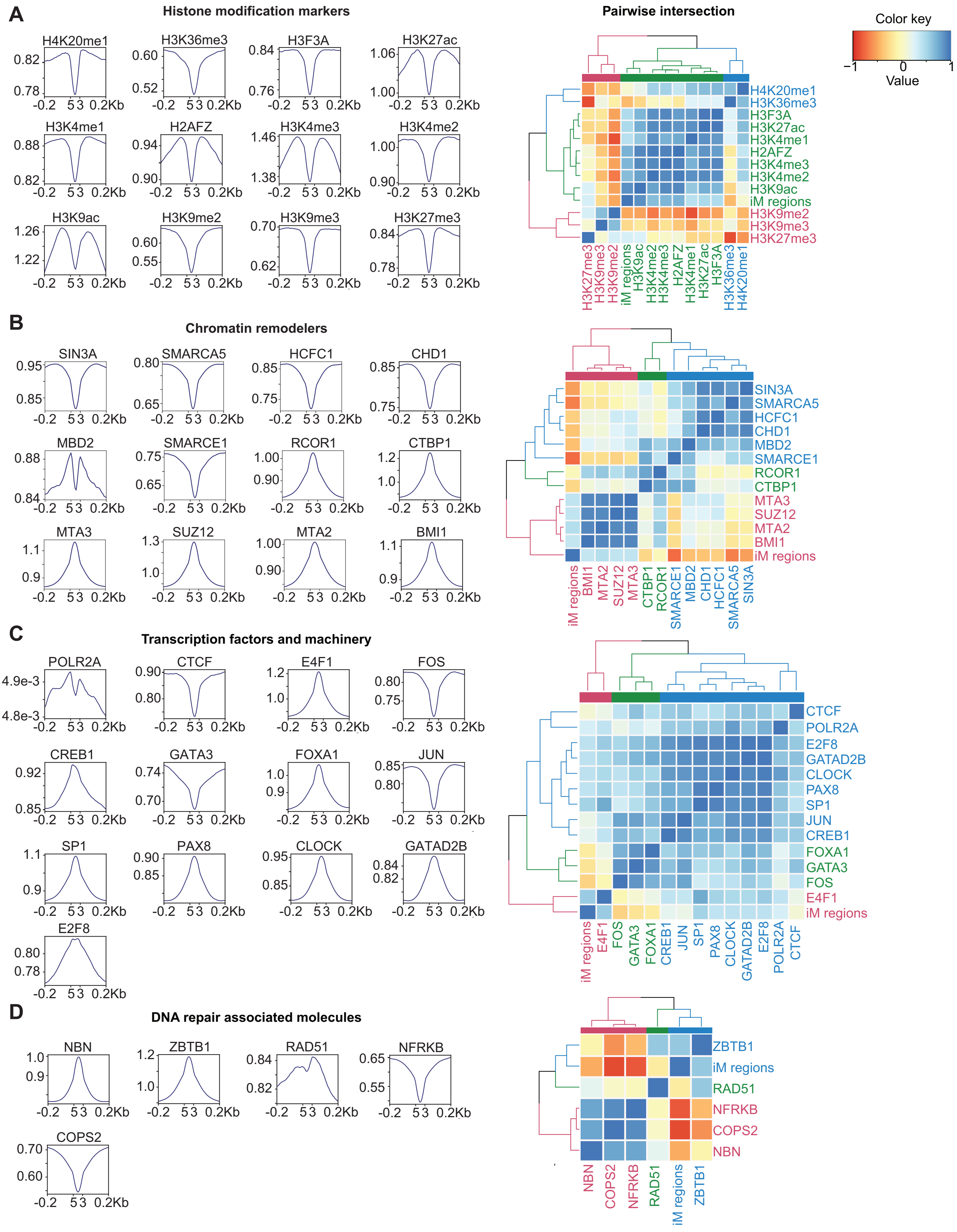
A
Histone modification markers
H4K20me1
H3K36me3
H3F3A
H3K27ac
H3K4me1
H2AFZ
H3K4me3
H3K4me2
H3K9ac
H3K9me2
H3K9me3
H3K27me3
Pairwise intersection
Color key
−1 0 1
Value
iM regions
B
Chromatin remodelers
SIN3A
SMARCA5
HCFC1
CHD1
MBD2
SMARCE1
RCOR1
CTBP1
MTA3
SUZ12
MTA2
BMI1
C
Transcription factors and machinery
POLR2A
CTCF
E4F1
FOS
CREB1
GATA3
FOXA1
JUN
SP1
PAX8
CLOCK
GATAD2B
E2F8
D
DNA repair associated molecules
NBN
ZBTB1
RAD51
NFRKB
COPS2
-0.2 5 3 0.2Kb

Figure EV4. iM regions occupy transcription machinery and active transcription histone modification sites.

Read count occupancy of iMab pulldowns relative to reported ChIP-sequencing from transcription factors and histone modifications in MCF7 cells (ENCODE) and pairwise intersection of iM regions in MCF7 cells (spearman correlation values shown). (**A**) Histone modification markers. (**B**) Chromatin remodelers. (**C**) Transcription factors and transcription-related machinery. (**D**) DNA repair-associated molecules.

 © The Author(s)

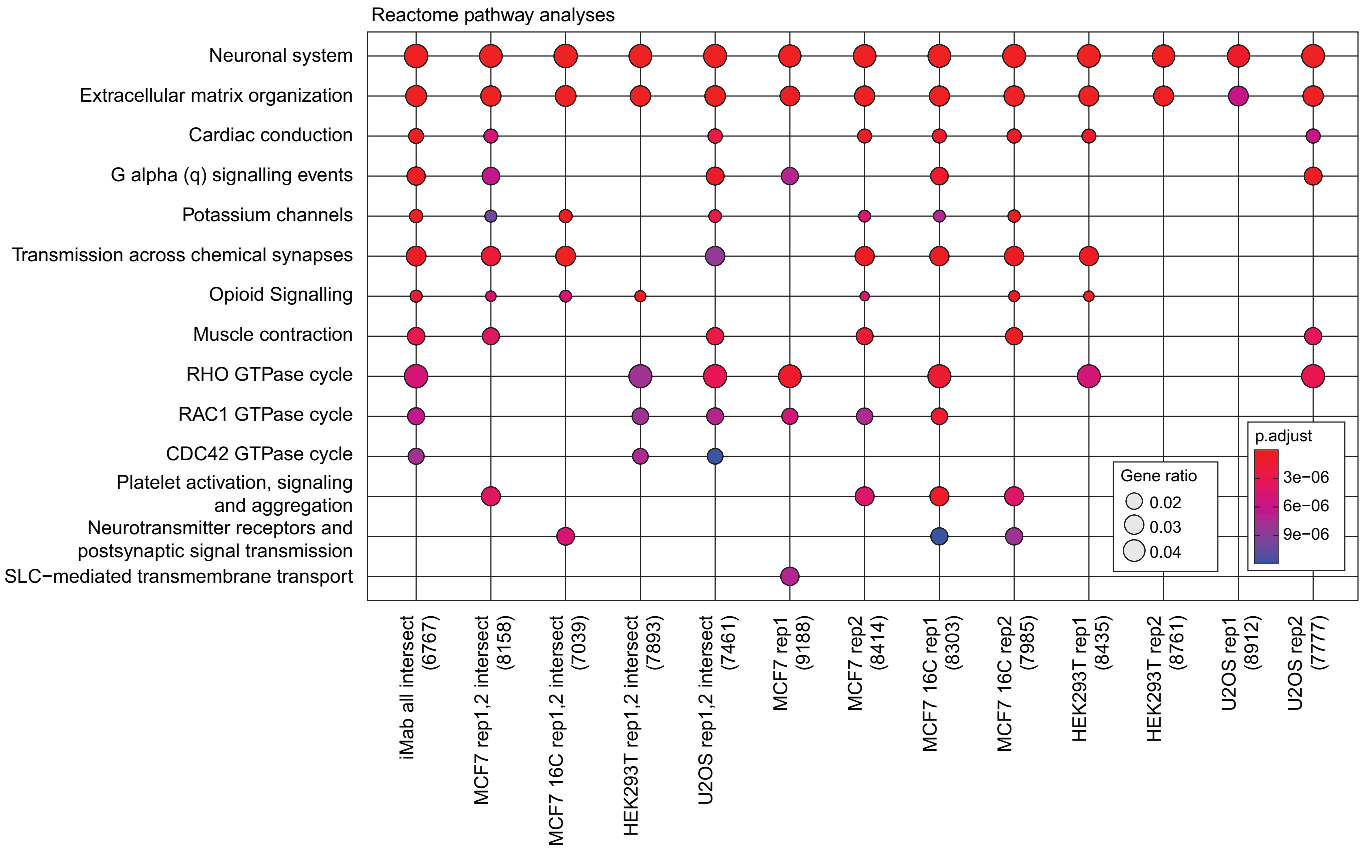

Figure EV5. Pathway enrichment across sample sets.

Reactome pathway enrichment analysis across samples from the different cell-lines and conditions. Ten most significant pathways shown with *P* value adjusted and scaled to highlight ratio of genes.

© The Author(s)
